# Temporal–Spatial Variations in Microbial Diversity and Community Composition in Surface Waters and Bottom Sediments of the Bohai Sea, China

**DOI:** 10.3390/microorganisms13092100

**Published:** 2025-09-09

**Authors:** Zhongyuan Li, Ying Yang, Yan Sun, Liang Zhao, Xianbin Liu

**Affiliations:** 1 Key Laboratory of Industrial Fermentation Microbiology of Ministry of Education, College of Biotechnology, Tianjin University of Science and Technology, Tianjin 300457, China; lizhongyuan@tust.edu.cn (Z.L.); gltosy@163.com (Y.S.); 2 College of Marine and Environmental Sciences, Tianjin University of Science and Technology, Tianjin 300457, China; yangying@163.com (Y.Y.); zhaoliang@tust.edu.cn (L.Z.)

**Keywords:** Bohai Sea, high-throughput sequencing, microbial community composition, environmental factors

## Abstract

Temporal variation in microbial communities is critical for sustainable utilization of marine biological resources and ecological restoration. However, microbial communities in China’s Bohai Sea remain inadequately characterized. This study employed high-throughput sequencing to characterize temporal–spatial variations in microbial communities within surface water and bottom sediment between June and August. A distinct temporal zone emerged in the sediments in August, characterized by low dissolved oxygen, bottom water acidification, and elevated concentrations of TN, NO_3_^−^, PO_4_^3−^, and DOC. Furthermore, temporal variation exerted a stronger influence on microbial community. August induced a significant decline in aerobic bacteria and an increase in anaerobes. Crucially, a substantial increase in ammonia-oxidizing bacteria (AOB) and nitrite-oxidizing bacteria (NOB), coupled with a decrease in denitrifying bacteria, likely contributed to observed NO_3_^−^ accumulation. Furthermore, complex alterations in sulfate-reducing bacteria (SRB) and sulfur-oxidizing bacteria (SOB) populations suggested potential impacts on sulfur cycling. This study provides critical insights for temporal–spatial variations in the microbial communities of the Bohai Sea, and provides a reference for developing effective coastal health indicators and further microbe resource exploration.

## 1. Introduction

Coastal marine environments are complex systems, which are not only affected by the interactions of geological, physicochemical, and biological factors but also largely influenced by anthropogenic activities, such as terrestrial pollution, aquaculture, and maritime transport [1]. Both surface water and bottom sediment are the crucial reservoirs for environmental pollutants including nutrients, antibiotics, and pesticides [2]. Organic matter mineralization and nutrient cycling are crucial for maintenance of coastal ecosystem health. Microorganisms in ecological systems can regulate and influence various biological and ecological processes, including carbon cycling, nitrogen fixation, organic matter decomposition, and sulfate reduction [3]. In recent years, the biodiversity and abundance of microbial communities and their major determining regulating factors are becoming important canonical indicators to predict the ecosystem responses to environmental changes and the extent of anthropogenic impact on coastal waters [4]. The various types of biodiversity of bacteria in different environmental ecosystems were previously explored, such as the surface water and/or bottom sediment of the Southern Ocean, Baltic Sea, Gulf of Mexico, and South China Sea [5]. The temporal–spatial dynamics of the biodiversity, abundance, and structure of a bacterioplankton community were found to be driven by various environmental factors including latitudinal gradient, temperature, and seasons nutrients [5]. Some dominant environmental factors could have shaped the corresponding microbial community, such as sulfate for sulfate-reducing prokaryotes, nitrogen content for denitrifiers, and salinity for halophilic microbes [6].

The Bohai Sea is China’s only semi-enclosed continental shelf marginal sea, and its marine ecosystem is largely affected by human activities and has faced immense pollution problems recently [7]. Previously studies have reported the biodiversity of associated bacterioplankton and phytoplankton in the coastal seawater of the Bohai Sea [8]. The microbial community is an important indicator for monitoring and resource exploration. To our best knowledge, only a few studies focused on the microbial community of the Bohai Sea. In 2006 and 2007, Li [9] investigated seasonal changes in bacteria in both the surface water and sediment of the Bohai Bay but did not establish a relationship with environmental factors. In terms of surface water in the Bohai Sea, Huang et al. [10] and Hu et al. [11] investigated the microbial diversity and community in August of 2014 and 2015, respectively. For sediment in the Bohai Sea, Lei et al. [12] studied the relationship between the microbial community and environmental factors in the Bohai Bay coastal sediments. Wang et al. [13] further compared the differences in microbial community composition, especially protease-producing bacteria, in the sediment of the Bohai Sea, Yellow Sea, and South China Sea in 2015. Thus, in the Bohai Sea, our current understanding of temporal–spatial variability in structuring microbial assemblages requires further investigation. The aim of this study was to comparatively investigate the biodiversity, abundance, and structure of bacterial communities in both surface seawater and bottom sediment of the Bohai Sea during June and August, as well as their relationship with environmental factors.

## 2. Materials and Methods

### 2.1. Sample Collection and Physicochemical Analysis

**Figure A1:**
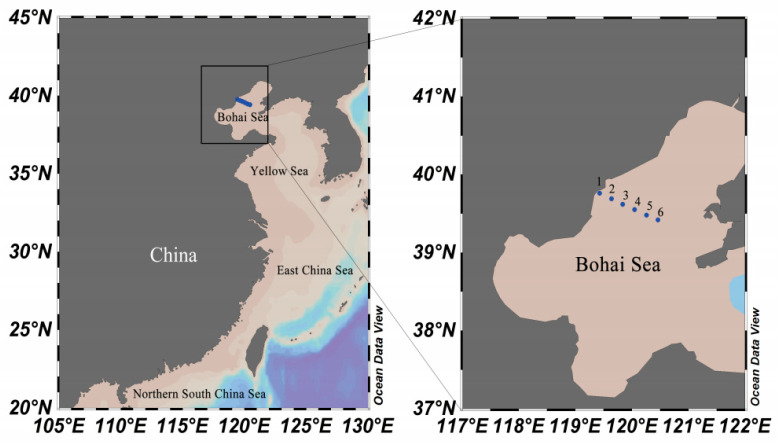
Study area and sampling sites in the Bohai Sea. Stations are designated by the number range 1 to 6.

**Figure A2:**
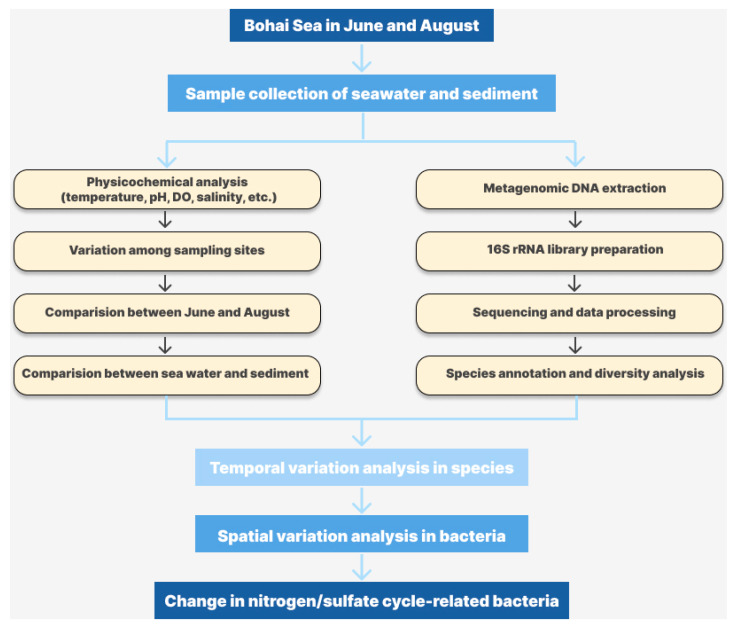
Schematic diagram of the physicochemical and microbial community analyses in this study.

Six sampling sites (stations 1–6) in this study were located along the coastal of Bohai Sea and close to the port and tourist city Qinhuangdao, which is situated about 280 km east of Beijing. The water and bottom sediment samples were both collected in the months of June and August in 2021 at stations 1, 2, 3, 4, 5, and 6 (Figure A1). Surface water (3 L) was collected from every station using a sample hydrophore, and the corresponding surface bottom sediment samples (1 kg) were collected by a box corer. The temperature, pH, DO, and salinity of the samples were measured by a portable YSI Pro Plus Multiparameter instrument (YSI Inc., Yellow Springs, OH, USA). The contents of PO_4_^3−^, Chlorophyll a (Chl a), total nitrogen (TN), ammonia nitrogen (NH_4_^+^), nitrate nitrogen (NO_3_^−^), nitrite nitrogen (NO_2_^−^), dissolved organic carbon (DOC), and dissolved inorganic carbon (DIC) in these samples were determined according to the “Specification for oceanographic survey” (GB/T 12763.4-2007) [14]. The physical–chemical parameters were all measured in replicates. A schematic diagram of the physicochemical analysis is shown in Figure A2.

### 2.2. Library Preparation and Sequencing

The surface water and bottom sediment samples for July and August were used to extract the metagenomic DNA for library preparation. The water sample (2 L) was filtered through a 0.22 μm membrane filter (AmeriTech Inc., Fort Lauderdale, FL, USA). All samples were packaged in sterile bags, transported immediately to the laboratory on dry ice and stored at −80 °C until DNA extraction. The metagenomic DNA of surface water (2 L) and bottom sediment (10 g) was extracted in replicates by the cetyltrimethylammonium bromide (CTAB) extraction method specifically designed for high-molecular-weight environmental DNA [15]. The extracted DNA was submitted to Novogene (Beijing, China) for further library construction and sequencing. The integrity, quantity, and quality of metagenomic DNA were measured by 1% agarose gels and NanoDrop ND-8000 (Thermo Fisher Scientific Inc., Waltham, MA, USA). The total metagenomic DNA was subjected to PCR amplification by targeting the 16S rRNA variable regions V4 using specific primers (515F: 5′-GTGCCAGCMGCCGCGGTAA-3′, 806R: 5′-GGACTACHVGGGTWTCTAAT-3′). The group without surface water and bottom sediment samples was used as the control group to eliminate potential contaminants. Sequencing libraries were generated by a TruSeq^®^ DNA PCR-Free Sample Preparation Kit (Illumina, San Diego, CA, USA). The library quality was measured on the Qubit^@^ 2.0 Fluorometer (Thermo Fisher Scientific Inc., Waltham, MA, USA) and Agilent Bioanalyzer 2100 system (Agilent Technologies Inc., Santa Clara, CA, USA). The barcoded library was then sequenced on an IlluminaHiSeq2500 platform to generate 250 bp paired-end reads.

### 2.3. Sequencing Data Processing and Statistical Analysis

The sequences were deposited in the Sequence Read Archive (SRA) with study accession SUB4631295. Effective tags were finally obtained after removing chimera sequences by the analysis of the UCHIME algorithm (UCHIME Algorithm). After sequence analysis by Uparse software (Uparse v7.0.1001), sequences with ≥97% similarity were assigned to the same OTUs. The taxonomic information of a representative sequence was annotated using the GreenGene Database based on RDP classifier (Version 2.2). All these indices in our samples were calculated with QIIME (Version 1.7.0) and displayed with R software v.3.3.1. Principal coordinates analysis (PCoA) for visualizing community dissimilarities (beta-diversity) was displayed by the WGCNA package, stats package, and ggplot2 package in R software (R v.3.3.1). The LDA (Least Discriminant Analysis) effect size was used to discover the taxa with significantly different relative abundances between different groups. A schematic diagram of the microbial community analysis is shown in Figure A2.

## 3. Results and Discussion

### 3.1. Geochemical Characteristics of Surface Water and Bottom Sediment Between June and August

**Figure 1:**
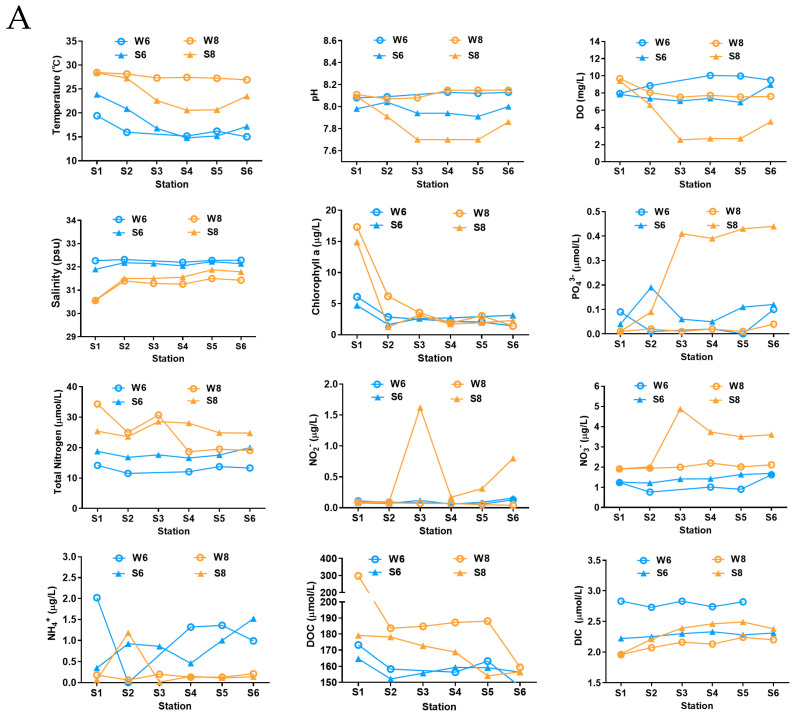

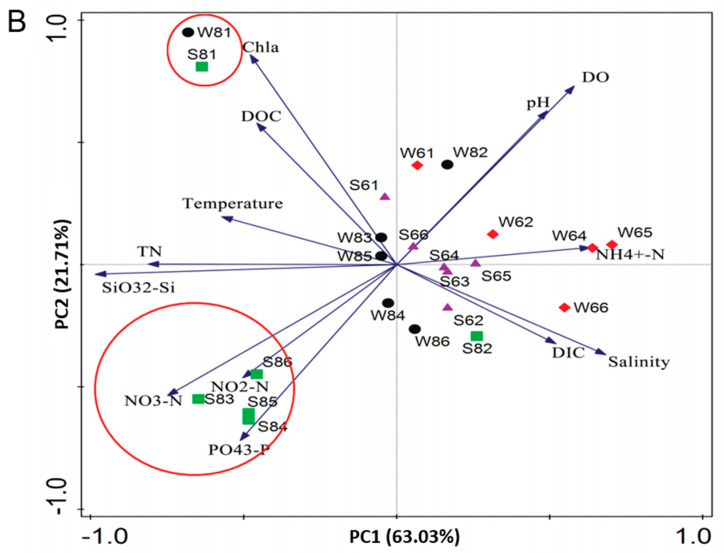
Physicochemical parameters of surface water and bottom sediment in the Bohai Sea. (**A**) The changed trends of physicochemical parameters. (**B**) Principal coordinates analysis (PCoA) presents the temporal and spatial variations of physicochemical parameters according to the weighted UniFrac distance and Bray–Curtis distance matrices. Water samples of June and August are labeled with red diamonds and black dots, and sediment samples of June and August are labeled with purple triangles and green squares. The hypoxic stations are marked by red circles.

The anticipated variation in the geochemical characteristics in the water and bottom sediment between June and August was observed (Figure 1A). From June to August, the average temperature of water increased from 16.36 °C to 27.59 °C, and the temperature of the bottom sediment also showed a slow increasing trend over time. It was also observed that the temperatures of the surface water in June at stations 1, 2, and 6 were lower than those of the bottom sediment, while in August, the temperatures of the surface water were much higher than those of the surface bottom sediment, especially in the off-shore stations (Figure 1A). The pH values of the surface water and bottom sediment in these three months were all alkalic, and the average pH of surface water (8.09) was a little higher than that of bottom sediment (7.89). Notably, the pH of bottom sediment at stations 3, 4, and 5 was dramatically decreased to 7.7 in August (Figure 1A). The DO concentration of the surface water was constantly higher than that of the surface bottom sediment, and a decrease was observed in the surface bottom sediment at stations 3, 4, and 5 in August with a DO concentration of only 2.55, 2.71, and 2.70 mg/L, which is close to hypoxia (2 mg/L).

For the nutritional salts, the change trends of concentrations of PO_4_^3−^, TN, NH_4_^+^, NO_2_^−^, NO_3_^−^, DOC, and DIC in surface water and bottom sediment were very complex in these two months (Figure 1A). The concentration of PO_4_^3−^ in surface water was higher than that of bottom sediment, and the concentration of PO_4_^3−^ in surface water showed a rapid rise, especially at stations 3, 4, 5, and 6, in August. Compared to June, the concentrations of total nitrogen (TN) in both the surface water and bottom sediment showed an increase in August, which was also reported previously in the Qinhuangdao area [6]. The concentration of NO_2_^−^ in the bottom sediment was stable at June and August, while the concentrations of NO_2_^−^ in the surface water at stations 3, 5, and 6 increased considerably in August. The concentration of NO_3_^−^ in the surface water increased gradually over time, and in the bottom sediment it increased from June to August, which shows the highest value at stations 3, 4, 5, and 6 in August. In contrast, the concentrations of NH_4_^+^ in the surface water and bottom sediment at these six stations all decreased in August. The concentration of DOC in the surface water and bottom sediment increased over time, whereas DIC decreased. The upper bottom sediment fractions also had higher concentrations of DOC during hypoxia, which may be related to higher rates of organic matter deposition on the bottom sediment with increased summertime phytoplankton production [16].

Furthermore, based on the large variations in geochemical characteristics among the near-shore and off-shore sites, the surface water and bottom sediment of the near-shore station 1 displayed the highest temperature, pH, DO, and Chl a, and were also abundant in nutrition concentration, indicating the large influence of human activity on the coastal marine. The PCoA analysis also showed that stations 3, 4, 5, and 6 were clustered together and showed a positive correlation with the concentration of PO_4_^3−^, NO_2_^−^, and NO_3_^−^, but a negative correlation with pH and DO (Figure 1B). Overall, by analyzing all the measured environmental variables in combination, the PCoA analysis showed distinct partitioning of surface water samples or bottom sediment samples across different months rather than stations (Figure 1B) [6].

### 3.2. Microbial Diversity and Community in Surface Water and Bottom Sediment

**Figure 2:**
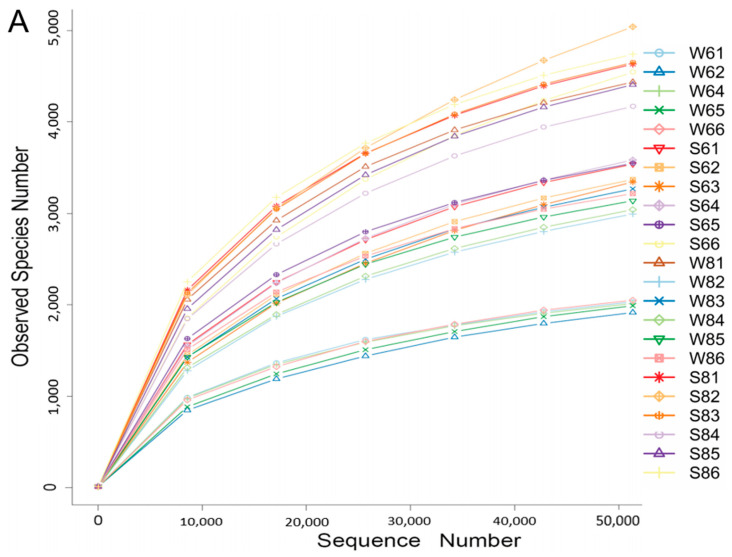

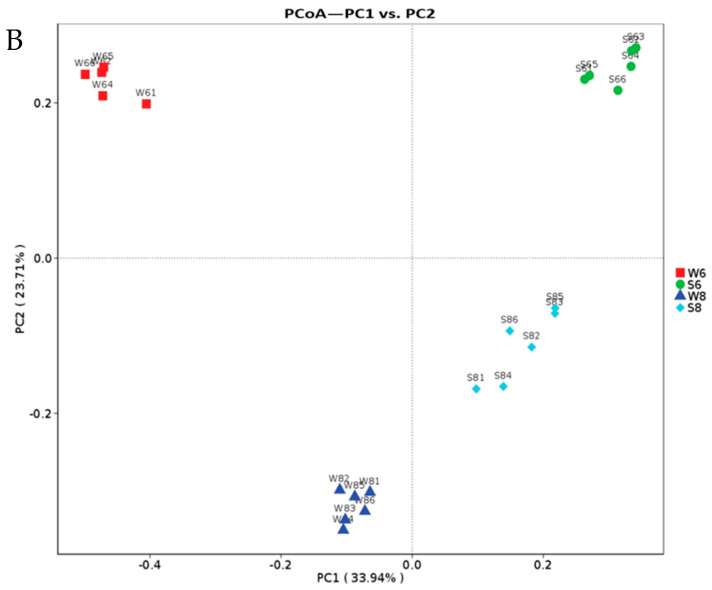
The microbial diversity of the surface water and bottom sediment in the Bohai Sea. (**A**) The rare curves of the surface water and bottom sediment. (**B**) Principal coordinates analysis (PCoA) presents the temporal and spatial variations in the bacterial community according to the weighted UniFrac distance and Bray–Curtis distance matrices.

The general diversity and community of bacteria in the surface water and bottom sediment of all sampling sites were assessed by 16S rRNA gene amplicon sequencing analysis, except sample W63, from which we failed to extract the genomic DNA. In total, 1,749,749 reads were generated from these 23 samples with HiSeq pyrosequencing. After quality control, 1,498,928 effective reads, nearly 85.67% of the total sequences were left for downstream analysis. A total of 97,314 Operational Taxonomic Units (OTUs) could be assigned at the 0.03 distance level by bioinformatic processing. The rarefaction curves of the original OTU table indicated that a large fraction of the diversity was reached at a depth of 50,000 reads (Figure 2A), and Good’s coverage values of all sites were all above 96%, suggesting that the 16S rDNA gene sequences obtained from these samples could represent most of the microbial community. Moreover, new microbial phylotypes continued to emerge, which suggests these samples contain abundant microbial resources.

The richness (Chao and ACE) and diversity (Shannon) of the bottom sediment were much higher than those of the surface water in both months, as shown in Table A1. In June, there were 5194 unique OTUs and 1278 unique OTUs in the bottom sediment (S6) and surface water (W6), respectively. In August, 3705 OTUs and 1768 OTUs were unique in the bottom sediment (S8) and surface water (W8). These results showed that 55.05 and 77.74% of shared microorganisms simultaneously exist in the surface water and bottom sediment, respectively. Moreover, the diversity of bacteria in the bottom sediment is much higher than that of the surface water. Furthermore, the OTU-based weighted UniFrac PCoA results (Figure 2B) showed that the surface water and bottom sediment were clustered together, which indicates that the prokaryotic community shows differences between the bottom sediment and surface water.

**Figure 3:**
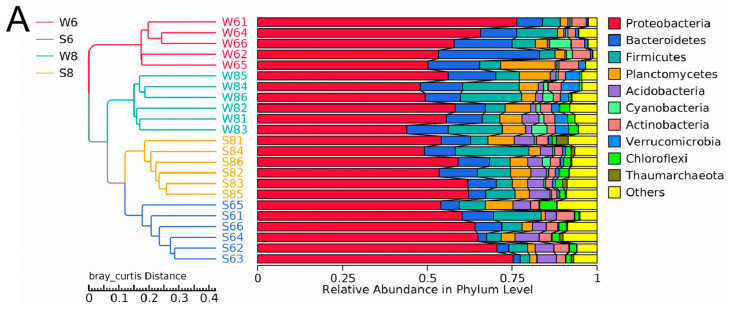

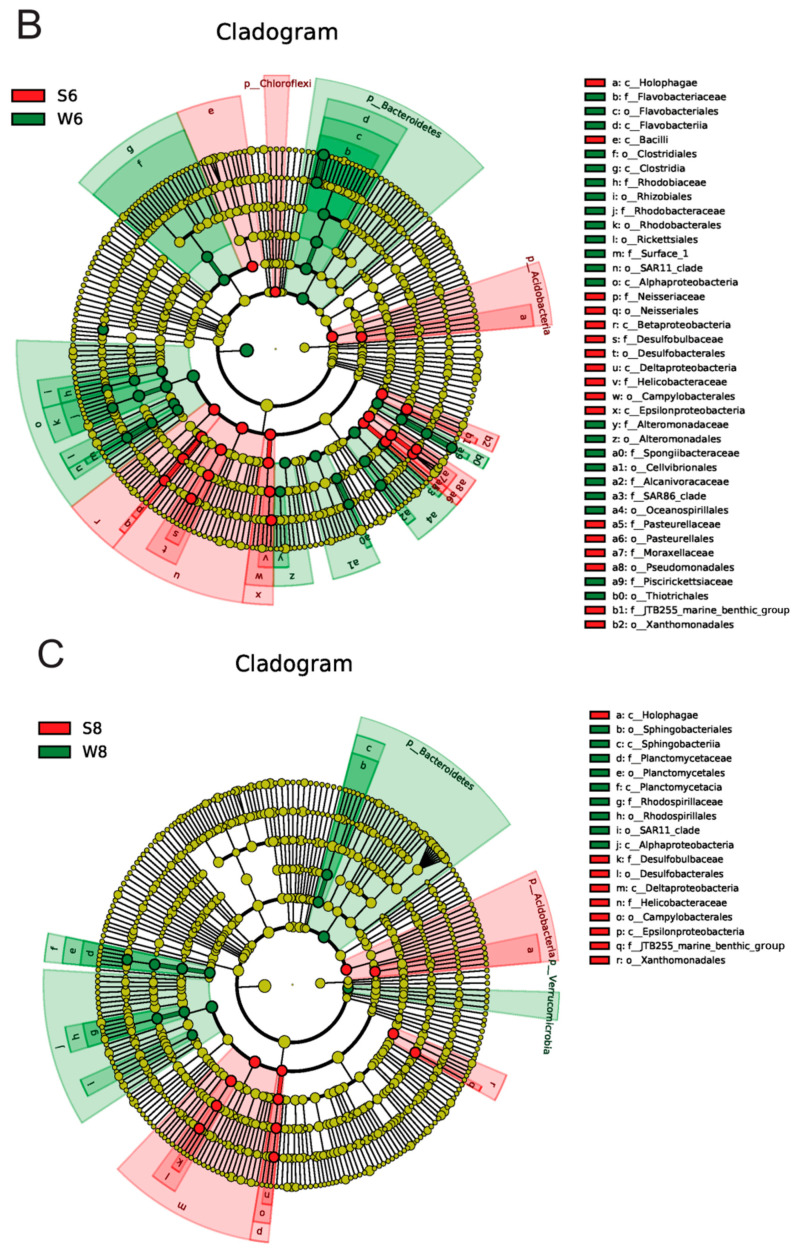

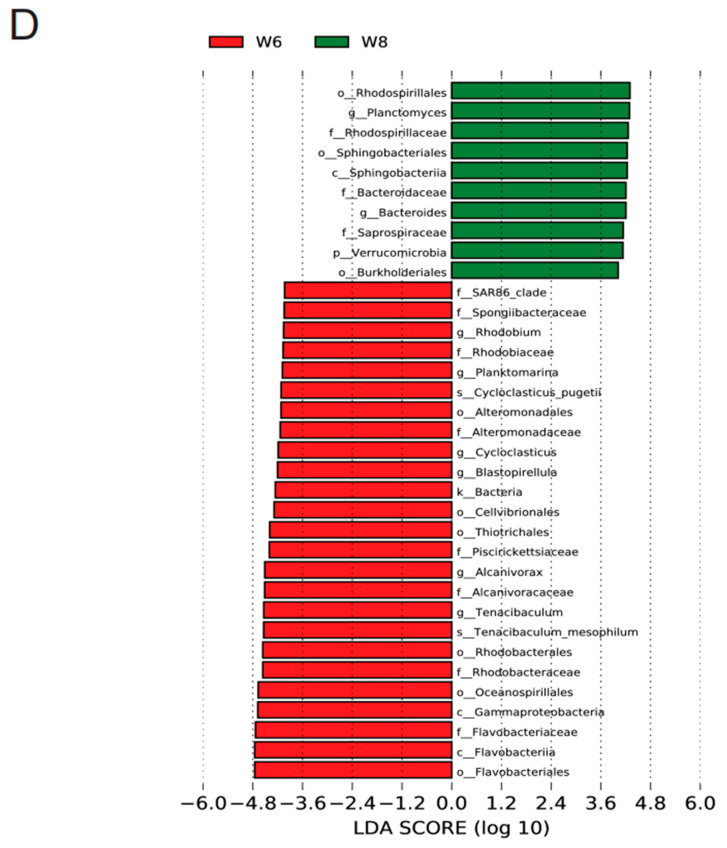

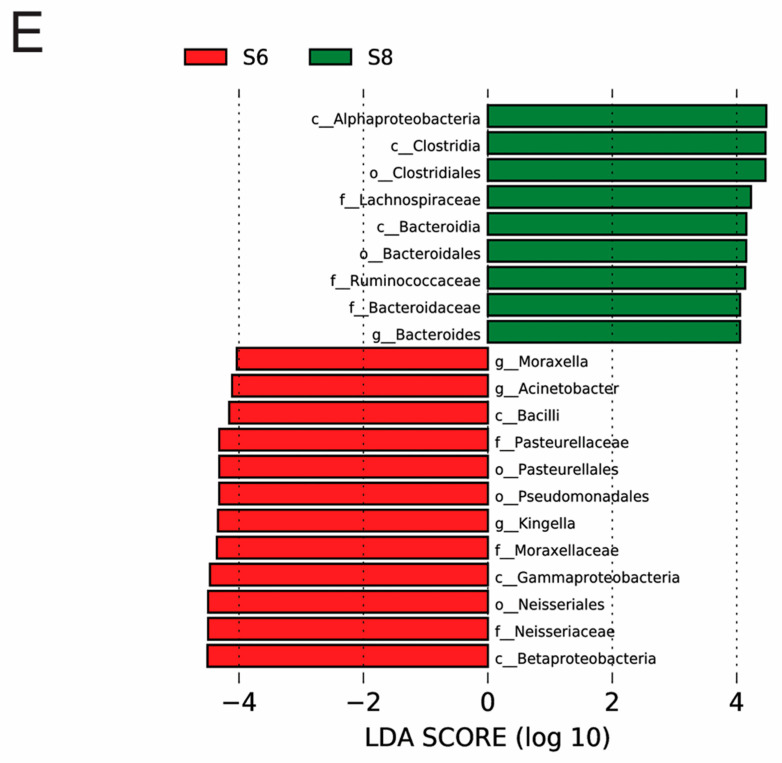
Dynamics of the bacterial community composition of the water and sediment samples from the Bohai Sea. (**A**) The dominant phylum in the sediment and water samples. Hierarchical clustering of samples based on the Bray–Curtis similarity algorithm. (**B**) Cladogram based on the LEfSe analysis of bacteria from phylum to genus level with relative abundance ≥1% between the surface water and bottom sediment in June. (Red) Sediment-enriched taxa; (green) water-enriched taxa. (**C**) Cladogram based on the LEfSe analysis of bacteria from phylum to genus level with relative abundance ≥1% between the surface water and bottom sediment in August. (Red) Sediment-enriched taxa; (green) water-enriched taxa. (**D**) Histogram of the LDA scores computed for features deferentially abundant between surface water of June and surface water of August. (**E**) Histogram of the LDA scores computed for features deferentially abundant between bottom sediment of June and bottom sediment of August. Enriched taxa for June are indicated with negative LDA scores (red), and enriched taxa for August are shown with positive scores (green). Only taxa meeting an LDA significant threshold of >3.6 are shown.

The microbial communities between the surface water and bottom sediment were distinct. At the phylum level, the highest relative abundance identified in the surface water of six sampling stations was assigned to *Proteobacteria* (51.88–60.69%), followed by *Bacteroidetes* (11.41–22.98%), *Firmicutes* (1.65–11.46%), *Planctomycetes* (1.88–6.61%), *Actinobacteria* (2.81–6.95%), and *Cyanobacteria* (1.95–4.79%) (Figure 3A). *Proteobacteria* and *Cyanobacteria* were previously found to be the most abundant phyla in the Qinhuangdao coastal waters. In particular, α-proteobacteria is usually abundant in coastal waters, which can form the predominant surface- and particle-colonizing group [16]. Moreover, the SAR11 clade, a subgroup of α-proteobacteria, was abundant in surface water in both June and August (Figure 3B,C). A previous study has also confirmed that SAR11 bacteria are abundant in marine environments, often accounting for 35% of total prokaryotes in the surface water of the ocean [17]. The SAR11 clade has highly depth-specific distributions, for example, SAR11 subclade II, SAR11 subclade Ia, and SAR11 subclade Ib were more prevalent in the upper mesopelagic, euphotic zone, and surface water, respectively [17]. It can adapt to nutrient-poor environments such as the surface water of the open ocean, an inability to reduce sulfate to sulfide, a requirement for pyruvate, and an unusual form of conditional glycine auxotrophy [17]. However, in the bottom sediment of the six sampling stations, *Proteobacteria* was also the predominant phylum, with a percentage between 56.63% and 64.86%, and the others were assigned to *Firmicutes* (3.49–9.34%), *Acidobacteria* (4.92–5.11%), *Bacteroidetes* (5.10–8.67%), *Actinobacteria* (2.36–6.95%), *Planctomycetes* (3.49–5.13%), and *Chloroflexi* (1.92–3.39%) (Figure 3A). *Proteobacteria* are reported to be predominant in surface water in sea environments such as the Yellow Sea and South China Sea, and estuary environments [13,18]. In contrast to the surface water, Acidobacteria, Chloroflexi, and Thaumarchaeota were abundant in the bottom sediment. In particular, some genera such as *Desulfobulbus*, *Sulfurovum* JTB255_marine_benthic_group, *Holophagae*, and *Xanthomonadales* were abundant in the bottom sediment (Figure 3C). *Sulfurovum* JTB255_marine_benthic_group was also highly represented in the South China Sea [13].

### 3.3. Variation in Microbial Diversity and Community Between June and August

As shown in Table A1, the OTU number of the surface water in August was much higher than that of June. Only 2762 OTUs were shared between June (W6) and August (W8), and there were 1374 and 5182 unique OTUs in June and August. Combining the increased Shannon and other indices suggested that the microbial diversity in surface water increased over time (Table A1). The OTU numbers of seawater and sediment in summer are also more than those in spring, autumn, and winter [9]. Moreover, the microbial community in surface water also varied from June to August (Figure 2B). At the phylum level (Figure 3A), the large contribution of Proteobacteria to the microbial community was constantly predominant in these two months. Bacteroidetes, Actinobacteria, Firmicutes, and Planctomycetes decreased in August, while Acidobacteria and Chloroflexi largely increased in August. The LEfSe analysis results (Figure 3D) further showed that Flavobacteriales, Rhodobacterales, Alteromonadales, Cellvibrionales, Oceanospirillales, and Thiotrichales were the representative strains in June, and Bacteroidaceae, Sphingobacteriales, Burkholderiales, and Rhodospirillaceae were the representative strains in August.

**Figure 4:**
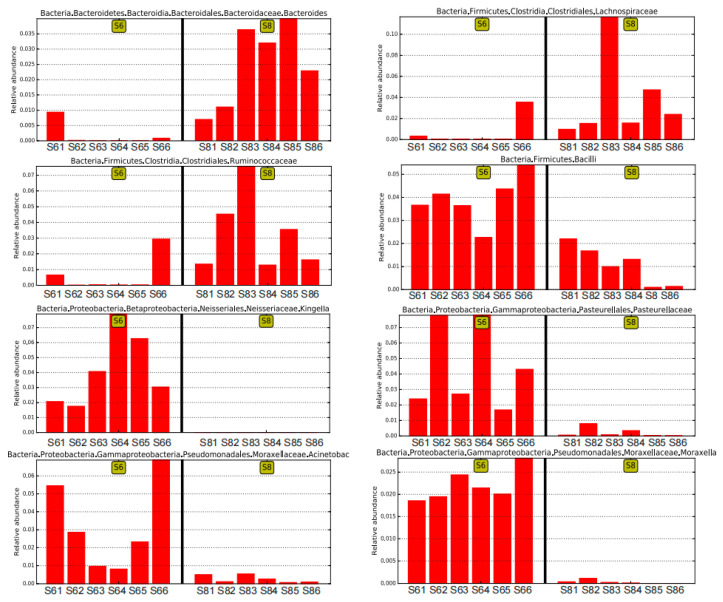
The changes in some representative bacteria in sediment of June and July based on the LEfSe analysis.

The variation in the microbial community was also observed in the bottom sediment between the two months. The average OTU number of the bottom sediment was 8502 in June (S6), and it increased to 9881 in August (S8), and 6025 identical OTUs were shared in the bottom sediment between June and August, which is consistent with the change trends of Shannon and other diversity indices. Furthermore, there were large differences in microbial community and abundance of bottom sediment between June and August (Figure 2B). At the phylum level, Proteobacteria, the major bacteria in bottom sediment, decreased in August, and it has been widely reported that Gammaproteobacteria strongly contribute to microbial population in many marine bottom sediment surfaces [19]. The main predominant phyla Planctomycetes, Firmicutes, and Bacteroidetes all increased over time, while Actinobacteria decreased in August (Figure 3A). Some bacteria like Bacilli, Neissseriales, Pasteurellales, and Pseudomonadales were abundant in the bottom sediment in June, and Bacteroidales, Clostridiales, and Alphaproteobacteria were abundant in August (Figure 3E). Among these bacteria (Figure 4), Acinetobacter was strictly aerobic, which is an important soil organism and contributes to the mineralization of aromatic compounds [20]. Moraxella is also aerobic bacteria, which means they could not survive in low DO conditions [21]. Moreover, Bacteroides increased dramatically in August, which might be because it is a genus of Gram-negative and obligate anaerobic bacteria. Lachnospiraceae and Ruminococcaceae belonging to the order Clostridiales both increased in S83, S84, and S85, because they are obligate anaerobes. Kingella (belong to Betaproteobacteria) decreased considerably, which might be because it is a species of Gram-negative aerobic bacteria [22] (Figure 4). A strong effect of oxygen on microbial community structure has recently been reported for pelagic ecosystems exposed to low oxygen concentration changes, and the low concentration of DO is reported to have severe negative impacts on other marine ecosystems [23]. In this study, the DO concentrations of stations 3, 4, and 5 (2.55, 2.71, and 2.70 mg/L) in August are close to hypoxia (<2 mg/L). Although these DO concentrations cannot be recognized as hypoxia (<2 mg/L), the increase in some anaerobes and the decrease in some aerobic bacteria might imply their potential relationship. Thus, it is necessary to continuously strengthen environmental regulation to prevent environmental deterioration.

### 3.4. Temporal Change in Nitrogen/Sulfate Cycle-Related Bacteria

**Figure A3:**
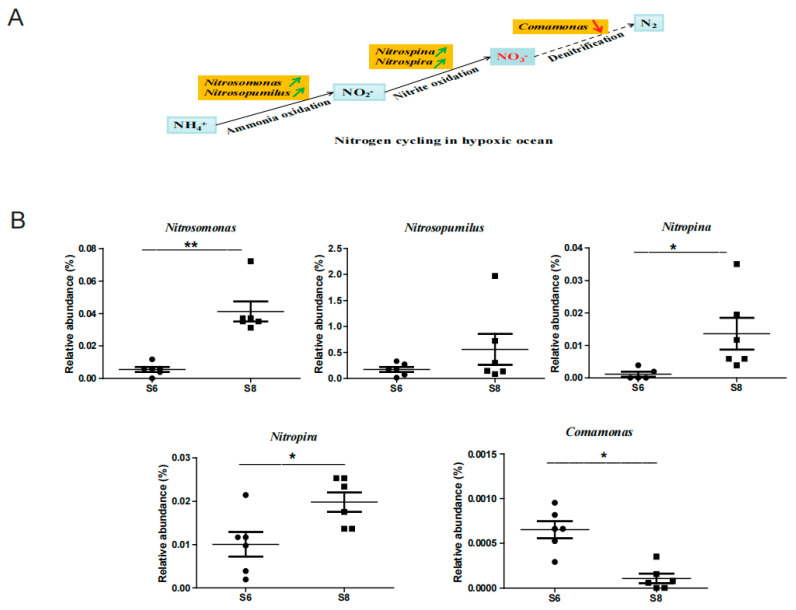
Impacts on the nitrogen cycle-related bacteria of Bohai Sea in the summer. (**A**) Partial nitrogen cycle in the sea and the typical related ammonia oxidation bacteria (AOB), nitrite-oxidizing bacteria (NOB), and denitrifying bacteria in this study. Green and red arrows represent the increasing and decreasing trend of relative abundance of bacteria. (**B**) Dynamics of the relative abundance of the nitrogen cycle-related bacteria from June to August. * represents *p* < 0.05, ** represents *p* < 0.01.

The concentration of NO_3_^−^ largely increased in samples S83, S84, and S85, but the concentration of NH4^+^ declined (Figure 1A), which might indicate the enhancement of nitrification for transforming NH_4_^+^ to NO_3_^−^. The nitrification rates are reported to be controlled by numerous factors including concentrations of NH_4_^+^, O_2_, temperature, and salinity, and especially by microbial communities [24]. The nitrification procedure is generally dominated by two kinds of microorganisms, namely, ammonia oxidation bacteria (AOB) and nitrite-oxidizing bacteria (NOB), which are responsible for the oxidation of ammonia to nitrite, and further to nitrate [21]. In this study, Nitrosomona belonging to AOB was found to increase in August (Figure A3), which is responsible for transforming NH_4_^+^ to NO_2_^−^ [25]. Nitrospina and Nitrospira belonging to NOB were also found to increase significantly in August (Figure A3). These two bacteria were reported to has the ability to transform NO_2_^−^ to NO_3_^−^ [26]. In addition, Comamonas, the typical denitrifying bacteria, decreased in August (Figure A3), and is reported to decrease in anoxic zones among the biological nitrogen removal process [25]. Consequently, the increase in AOB and NOB, and the decrease in denitrifying bacteria might be one of the reasons for the accumulation of NO_3_^−^ in August in the Bohai Sea. Combined, the changes in the representative bacteria and the nutrient salts provide a novel snapshot of the nitrogen cycle of the Bohai Sea between June and August.

**Figure A4:**
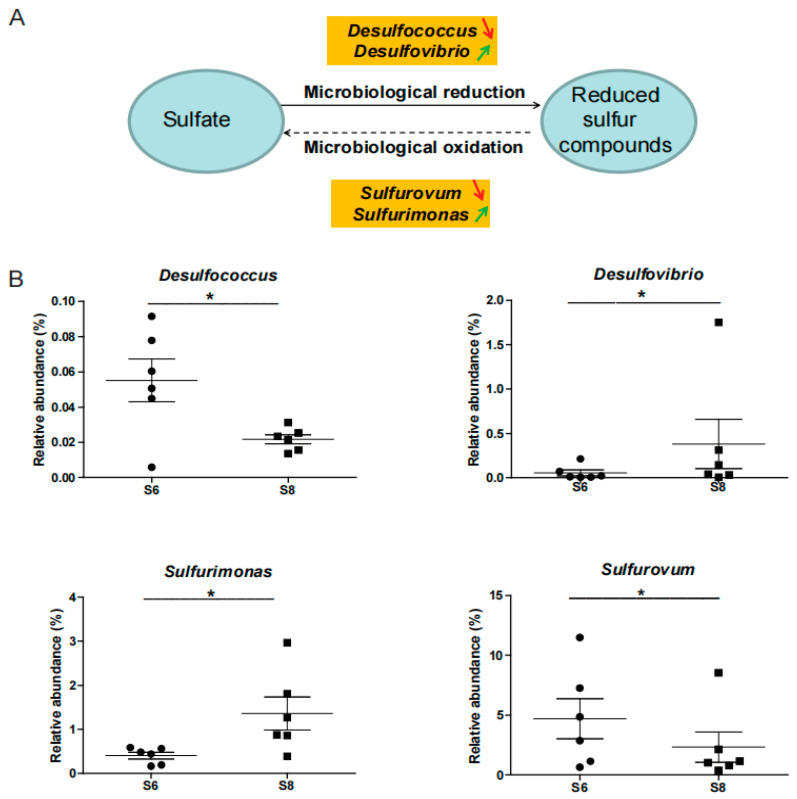
Impacts on the sulfate cycle-related bacteria of the Bohai Sea in the summer. (**A**) Microbiological reduction and oxidation-related bacteria in the sea. Green and red arrows represent the increasing and decreasing trend of relative abundance of bacteria. (**B**) Dynamics of the relative abundance of the sulfate cycle-related bacteria from June to August. * represents *p* < 0.05.

The community of the sulfate-reducing bacteria (SRB)-related strain significantly changed between June and August in this study. The abundance of genus Desulfovibrio increased from June to August, and the genus Desulfococcus decreased (Figure A4). These results showed that different SRB groups exhibit different trends between spring and summer, which might be because different types of substrates support the growth of specialized groups of SRB [27]. Moreover, although SRB in organic-rich coastal bottom sediment produce a large amount of hydrogen sulfide, most of this toxic metabolite could be re-oxidized at the bottom sediment–water interface by sulfur-oxidizing bacteria (SOB) [28]. SOB such as Sulfurovum and Sulfurimonas were reported to be abundant in the hypoxia treatments during a 28-day microcosm experiment of surface bottom sediment in the Baltic and Black seas [23]. In this study, the abundance of Sulfurovum decreased and Sulfurimonas increased from June to August (Figure A4). These results suggested that these bacteria might be important in controlling the nitrification and sulfate cycle in the Bohai Sea. Thus, the changes in nitrification and sulfate cycle-associated bacteria require further monitoring, while the potential function of these bacteria in bottom sediment needs verification in further study.

## 4. Conclusions

In summary, this study initially examined the temporal–spatial dynamics of environmental parameters in the Bohai Sea across June and August. The sediments at August formed a distinct cluster characterized by low oxygen, acidification, and elevated nutrients. The microbial community differences between June and August were further analyzed by high-throughput sequencing. Temporality shaped microbial communities, reducing aerobic bacteria and increasing anaerobes in sediment. Shifts in ammonia-oxidizing, nitrifying, and denitrifying bacteria might have impacted nitrogen cycling. This study provides a reference for subsequent research on microbial resources and environmental protection in the Bohai Sea. Nevertheless, limited time point samples were selected in this study, and more detailed experiments should be implemented in subsequent research, for example, on the seasonal change in microbial communities as well as the potential function of the specific strains in coastal ecosystems.

## Data Availability

The original contributions presented in this study are included in this article; further inquiries can be directed to the corresponding author.

## Appendix A

**Table A1:** The richness and diversity indices of the surface sediment and water.

Sample Name	OUT Number	Shannon	Simpson	Chao1	ACE	Goods_ Coverage	PD_Whole_Tree
W61	2505	7.904	0.987	2437.02	2543.913	0.989	142.16
W62	2441	6.724	0.951	2433.088	2675.738	0.987	137.261
W64	2459	7.523	0.981	2440.64	2572.292	0.989	142.991
W65	2601	6.83	0.967	2425.324	2646.712	0.987	143.777
W66	2570	7.307	0.977	2553.8	2666.737	0.988	144.284
S61	4448	8.787	0.991	4134.812	4540.36	0.979	219.542
S62	4205	8.305	0.985	4199.19	4391.863	0.979	205.413
S63	4322	7.691	0.971	4757.214	4931.703	0.974	205.154
S64	4507	8.434	0.988	4359.483	4651.167	0.978	218.714
S65	4360	8.754	0.99	4102.951	4304.009	0.982	218.033
S66	5723	9.164	0.993	6282.091	6420.728	0.966	273.959
W81	3781	9.602	0.995	5207.137	5361.166	0.977	286.45
W82	3719	7.979	0.984	3769.446	3968.268	0.981	206.382
W83	3419	8.332	0.983	4067.308	4230.991	0.98	224.74
W84	3271	8.318	0.985	3834.126	4033.948	0.98	206.487
W85	4037	8.553	0.99	3834.873	3971.655	0.982	219.648
W86	4078	8.844	0.993	3767.456	3926.313	0.983	236.14
S81	6238	9.928	0.996	5530.864	5730.346	0.975	270.608
S82	5850	9.783	0.996	7588.229	7702.544	0.96	297.919
S83	6251	9.487	0.99	5445.14	5676.138	0.975	287.929
S84	6063	9.197	0.992	4998.356	5231.917	0.976	267.312
S85	5878	9.449	0.994	5298.321	5589.625	0.974	272.243
S86	5776	9.97	0.996	5431.161	5637.909	0.977	294.433
